# Empirical Evidence for the Outcomes of Therapeutic Video Games for Adolescents With Anxiety Disorders: Systematic Review

**DOI:** 10.2196/games.9530

**Published:** 2018-02-28

**Authors:** Steven Barnes, Julie Prescott

**Affiliations:** ^1^ Bury College Bury United Kingdom; ^2^ Department of Education and Psychology University of Bolton Bolton United Kingdom

**Keywords:** anxiety disorder, video games, adolescent, CBT, eHealth, mental health, mobile health

## Abstract

**Background:**

Extant evidence suggests that the proportion of adolescents suffering from anxiety disorders (ADs) has increased by up to 70% since the mid-1980s, with experience of anxiety at this stage associated with significant negative short- and long-term life outcomes. The existing therapeutic interventions (eg, cognitive behavioral therapy, CBT; attention bias modification, ABM) have proven to have clinically measurable benefits in reducing anxiety, but their efficacy is often compromised by social and practical barriers. The growing discrepancy between demand for, and access to, clinical interventions for anxiety has led to the development of a range of eHealth (health care practice supported by electronic processes and communication) and mHealth (versions of eHealth using mobile devices) interventions. One such protocol is therapeutic games, which aim to provide clinical frameworks in dynamic, adaptable, and personalized virtual environments. Although some evidence exists to suggest therapeutic games are associated with reductions in subjective anxiety and observed stress reactivity, there is currently, to our knowledge, no systematic review of the adherence to, and effectiveness of, therapeutic games for adolescent anxiety.

**Objective:**

The aim of this review was to establish the effectiveness of therapeutic games in making clinically measurable reductions in anxiety symptoms in adolescent samples.

**Methods:**

A systematic search of the existing academic literature published between 1990 and July 2017 was conducted using the databases Journal of Medical Internet Research, Journal Storage, Psychology Articles, Psychology Info, ScienceDIRECT, and Scopus. Records linked to empirical papers on therapeutic games for anxiety using adolescent samples were evaluated.

**Results:**

A total of 5 studies (N=410 participants) met the inclusion criteria, and 3 gamified anxiety interventions for adolescents were identified. The papers included a mixture of randomized controlled trials, quasi-experimental studies, and usability studies comprising quantitative and qualitative measures, with varying degrees of mixed methods. Extant evidence shows potential for therapeutic games to create clinically measurable reductions in symptoms of anxiety in adolescent samples, though findings are complicated in some cases by a low sample size, and in other cases by research design and methodological complications, including anxiety reductions in control groups caused by a control-game selection.

**Conclusions:**

Although research in this field appears to be extremely limited, as demonstrated by the small number of papers meeting the inclusion criteria for this review, early findings suggest that therapeutic games have potential in helping to engage adolescents with anxiety and lead to clinically measurable reductions in symptoms.

## Introduction

### Anxiety Disorders and Adolescence

The term anxiety disorder (AD) represents a category of psychological disorders characterized by feelings of anxiety about future events, and fear reactions to current events [1], in addition to increased attentional biases toward threat detection [2]. ADs are the most prevalent of the psychiatric disorders [3], affecting approximately 117 million young people worldwide; it is the sixth leading cause of disability, with the largest longevity among young people aged 15-34 years [4], with a range of factors such as misdiagnosis, health care avoidance behaviors, and hardiness, meaning these statistics change relentlessly.

The extant evidence suggests that the proportion of adolescents suffering from ADs has increased by up to 70% since the mid-1980s and that nearly 300,000 young people in the United Kingdom have a diagnosable AD [5]. The onset of AD increases significantly during the adolescent years [6], in part as a result of conflicts regarding existential identity [7], educational pressures and high self-expectations [8], negative peer comparisons or perceived relational victimization [9], and over-demanding intrusive parenting [10,11]. Experience of AD in early life is associated with negative short- and long-term implications for social, academic, financial, and health performance [12] and predicts adult anxiety and substance abuse disorders [13].

For the individual, adolescence is both a source of increased opportunity and increased pressure and risk [14]. Increased social expectations of developing autonomy in self-regulation and self-determination of behavior, coinciding with diminishing assistance from adults, require the adolescent individual to develop and coordinate effective emotional and cognitive capabilities in relatively short time frames. These time frames, however, do not always correlate well with progress made in brain maturation [15,16]. Functional magnetic resonance imaging data also point to a tendency toward an increased response to emotionally loaded stimuli at this age [17,18]. Neuroimaging data point to a biomaturational explanation for the increased prevalence of AD in the adolescent years [19]. Furthermore, naturally occurring consolidation of neural pathways during adolescence may explain the tendency of experience of AD at this age to lead to negative outcomes in later life, leading some to describe AD as a potential gateway disorder [20]. Therefore, effective treatment of adolescent AD is critical in the mitigation of both its impact at the point of experience and the potential long-term ramifications [21].

### Therapeutic Interventions for Anxiety Disorders

Cognitive behavioral therapy (CBT) has been shown to be highly effective in the treatment of ADs [22], reducing or eliminating symptoms through the development of effective behavioral adjustment and coping strategy enhancement. Attention bias modification (ABM), an emerging technique derived from neurocognitive models of anxiety, has also been noted for having significant potential to enhance both pharmacological and psychological interventions for anxiety, as well as being an effective standalone intervention [23]. However, although evidence-based early intervention strategies reduce the probability of negative life outcomes [24], practical barriers to treatment (eg, cost) and social barriers to treatment (eg, stigma) mean as many as 50% of people in the United Kingdom experiencing anxiety do not seek treatment [25]. For those who seek treatment, waiting lists via Improving Access to Psychological Therapies (IAPT) referrals can be lengthy, leading to high dropout [26]. In addition, educational institutions and universities often fail to provide adequate support [27], with the existing services unable to meet the rising demand [28]. As a result, in adolescents, it is estimated that less than 20% of individuals affected by ADs receive treatment [29], with fewer than 20% of those seeking and receiving treatment being provided with interventions supported by scientific evidence [30].

The growing discrepancy between demand for, and available provision of, mental health services has led to the development of a range of alternative methods for delivering clinical interventions for anxiety [31]. eHealth (health care practice supported by electronic processes and communication) and mHealth (versions of eHealth using mobile devices) models (eg, computerized cognitive behavioral therapy) aim to mitigate the impact of both practical and social barriers to treatment by utilizing ubiquitous mediums to broaden the reach of clinical models [32-34]. One such medium is therapeutic video games, a derivative of serious games. Due to the improved realism in simulated artificial environments and capabilities of contemporary hardwares, Web-based therapies are more comparable than ever to in vivo forms of treatment [35]. The gamification of clinical models may be particularly suitable for younger people, as they often reflect the typically more visual, rapid, and multi-tasking learning styles of a generation with a lifelong exposure to and familiarity with technology [36,37]. Therapeutic games afford a flexible and personalized learning environment that allows for exploratory learning and behavior practice [38], allowing Web-based environments to be adapted in terms of content and challenge to the requirements of the user, which is likely to be conducive to an enhanced learning experience [39,40]. As games utilize both intrinsic and extrinsic motivational elements in active and realistic learning opportunities with immediate opportunities for feedback, they have already been shown to be capable of eliciting improvements in self-awareness and self-management behaviors in people with chronic physical health conditions [41]. In terms of the benefits of therapeutic games for mental health and well-being, the extant evidence suggests they may be effective across a variety of disorders, including reducing psychopathological symptoms associated with gambling disorders [42], and as an effective preliminary treatment to CBT for bulimia nervosa [43]. Although the current literature presents conflicting evidence regarding the health benefits versus health hazards of video game platforms [35,44], therapeutic games utilize a popular platform to achieve clinically measurable health improvements and behavioral changes [45].

### Research Questions

Therapeutic games provide young people with a dynamic, adaptable, and personalized learning environment in which they are afforded an opportunity to seek relevant information and guidance in an exploratory manner, receive immediate feedback, and utilize unlimited opportunities for repeat engagement [38]. As a result, therapeutic games should offer a more accessible platform for rapid learning and adoption of scientifically validated therapeutic techniques.

Although some evidence exists to suggest that therapeutic games can be linked with reductions in subjective anxiety and observed stress reactivity [46], research to date often combines adolescent samples with either child or adult participants, limiting the capacity of the current data in terms of its applicability to the unique nature of anxiety experienced at this life stage. Furthermore, to the researchers’ knowledge, there is currently no stated set of guidelines available for the development of therapeutic games, to which developers are required to adhere, nor is there a definitive protocol established for their scientific evaluation.

Consequently, it is unclear whether the potential benefits of therapeutic games establish themselves in anxiety in adolescents, and if so, whether any benefits are modulated by the therapeutic framework employed. A systematic review was conducted to assess the effectiveness of therapeutic games in enhancing engagement with clinical interventions, and their efficacies in making clinically measurable reductions in AD symptoms in adolescent samples.

## Methods

### Databases Searched

Relevant papers were identified by performing a comprehensive literature search of the following databases: Journal of Medical Internet Research, Journal Storage, Psychology Articles, Psychology Info, ScienceDIRECT, and Scopus.

### Search Terms and Selection of Papers for Inclusion

The following search terms were used to address the variety of games that might be played, and the variation in terms used to describe them: all (“serious game” OR “video game” OR “therapeutic game” OR “online game”) AND all(“adolescen*” OR “teenage” OR “youth” OR “young adult*”) AND “anxi*.” For further detail regarding search terms, definitions, and variation of input, see Multimedia Appendix 1.

Paper abstracts were initially scanned to determine eligibility. If eligibility could not be determined from the abstract alone, or if the paper was deemed as potentially relevant from the abstract, the full-text paper was studied for its relevance to the review.

### Inclusion Criteria

In line with the Preferred Reporting Items for Systematic Reviews and Meta-Analyses (PRISMA) guidelines, clear inclusion criteria were established to determine the eligibility of papers for inclusion in the review. Only studies meeting the following criteria were considered eligible for inclusion: papers linked to therapeutic games for ADs; studies conducted on adolescent samples; empirical research using a randomized controlled trial (RCT) design, quasi-experimental design, or correlational design; and studies using a control group or pre- versus posttest design to allow for comparison. All searches were limited to anxiety.

Adolescent sample was defined using American Psychiatric Association criteria (10-19 years). As a result, papers that used a sample of participants aged between 10 and 19 years were considered eligible. Papers that used samples including participants aged 8 or 9 years were considered eligible if the mean age for the sample was over or very close to 10 years.

Papers published in English or German were selected and subjected to the inclusion criteria as outlined above. In line with PRISMA guidelines, a specific date range was established. Studies published between January 1990 and July 2017 were selected. This date frame was chosen as papers first studying the effect of video games in the context of health education were published in the 1990s [41].

### Exclusion Criteria

Papers regarding opinion pieces, existing literature reviews, conference posters, and design documents for therapeutic games were excluded from the review. Study protocols were also eliminated from the review as they would be unable to provide outcome measures. Pilot studies were included in the review provided the 4 criteria discussed above were met.

### Quality Assessment

For the purposes of consistency, one researcher oversaw the initial coding of the papers. Quality assessment of papers meeting the inclusion criteria was assessed using the mixed methods appraisal tool (2011) [47]. The mixed methods appraisal tool is designed for systematic reviews, including a combination of quantitative, qualitative, and mixed methods studies, and has been noted for its reliability and efficiency as a quality assessment protocol, and capability to concomitantly appraise methodological quality across a variety of empirical research [48]. In line with PRISMA guidelines, an interrater process was adopted and the degree of agreement was assessed, to reduce risk of bias.

### Primary Outcome Measure

To assess the extent to which therapeutic games elicit reductions in AD symptoms, papers were studied for comparisons between measures of anxiety symptoms at pre- versus postintervention.

## Results

### Papers Meeting Inclusion Criteria

A total of 2259 records were identified through database searches. After papers published in languages other than English or German, and duplicate instances of papers were removed, remaining papers were assessed using the inclusion and exclusion criteria outlined above (N=2222). Initially, abstracts were searched to assess a paper’s eligibility for inclusion. If abstract information alone was not sufficient to determine whether a paper met the criteria, the entire paper was studied.

**Figure 1:**
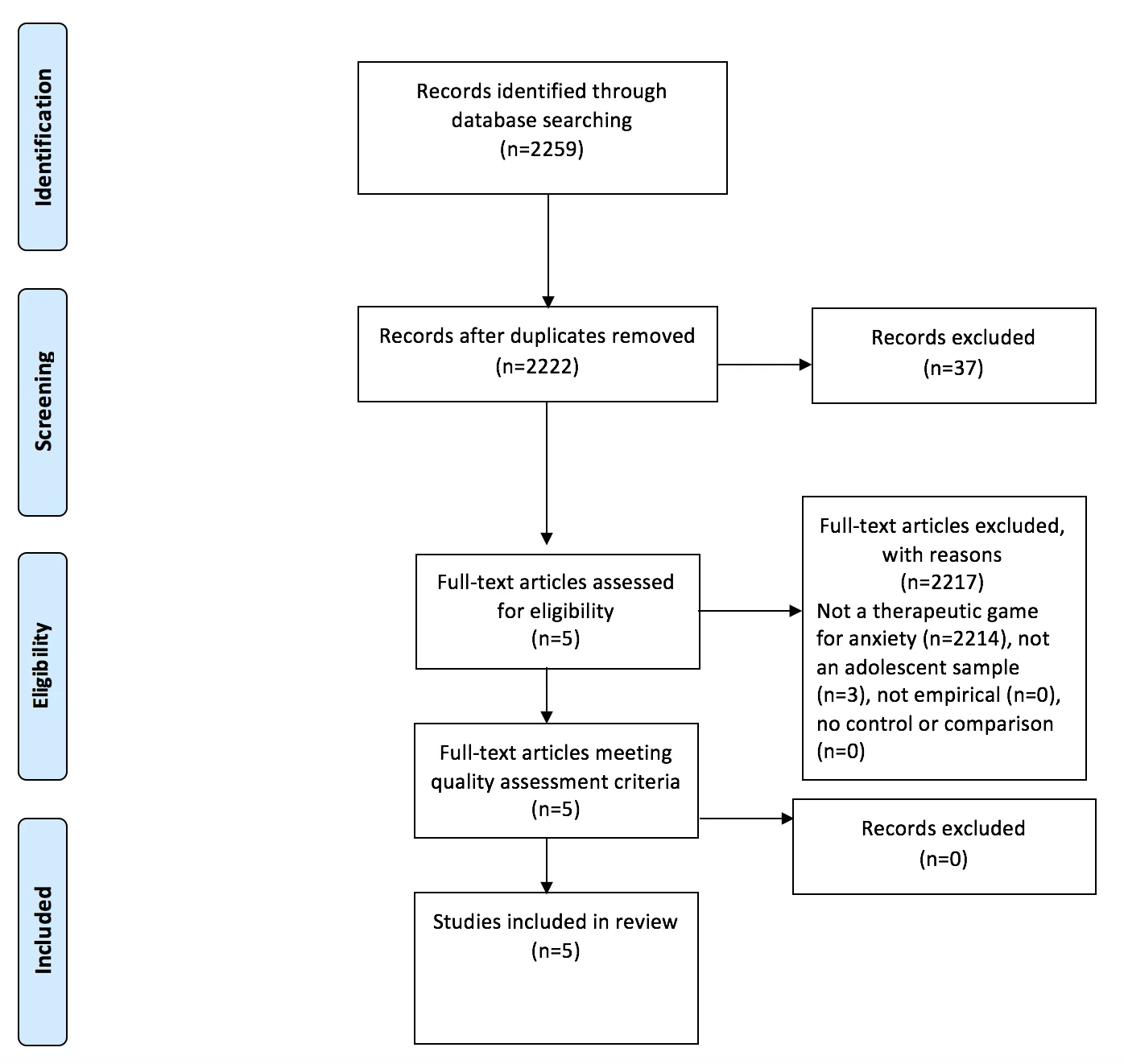
Outcome of literature search.

Figure 1 shows the number of academic papers from each database identified using the search terms and the number of papers meeting the inclusion criteria.

### Quality Assessment Outcomes

Papers meeting all of the inclusion criteria (N=5) were then quality assessed using the procedure described above. Subsequent to these papers meeting the quality criteria, they were included in the review. The mean rating for the papers was 75%, and the modal rating for the papers was 75%.

Owing to the small number of papers meeting the inclusion criteria, all papers included in the review were subsequently coded again for interrater reliability. The interrater reliability for the total scores was 0.8, showing good agreement between the 2 coders regarding paper quality.

### Overview of Papers Included in the Review

Of the 5 studies selected as meeting the criteria for inclusion in the review, 2 relied solely on quantitative measures [49,50] and 3 used a mixed-methods approach [51-53], with differing balances of reliance on quantitative versus qualitative data. Of the papers reporting quantitative data (N=5), 2 used an RCT design [49,52], 1 used a quasi-experimental design [50], and 2 were exploratory evaluations and usability studies [50,53]. Of the papers reporting qualitative data (N=3) [51-53], all used interview techniques to obtain varying amounts of qualitative feedback.

Of the 2 papers focusing on the therapeutic game “Dojo” [51,52], one study focused on a pilot study to evaluate perceptions and feasibility of the game [51], whereas the other paper concerned an RCT comparing the game to “Rayman 2: The Great Escape,” in terms of their relative abilities to reduce adolescent anxiety [52].

In terms of quantitative data, one paper focused on the neurofeedback game “MindLight” [49] using an RCT to compare the game to “Max and the Magic Marker” in terms of their relative abilities to reduce adolescent anxiety.

One paper used a mixed-methods approach to qualitatively evaluate impressions of the game “gNats Island” using a sample of 6 adolescents [53]. Pre- and posttreatment, and 6-week follow-up, measures of anxiety were assessed quantitatively using a range of clinical measures to indicate symptom levels for anxiety and a range of other mental health conditions.

The final paper included in this review utilized a quantitative approach to assess the efficacy of an augmented reality (AR) therapeutic game [50]. A quasi-experimental design was adopted for this study, recruiting participants to experimental (AR exposure therapy) and control (no game exposure) conditions in 2 separate phases.

### Outcomes of Therapeutic Games on Adolescent Anxiety

Schoneveld et al [49] utilized an RCT design to examine the anxiety-reduction effects of the game “MindLight”—a game designed for children and adolescents using a combination of CBT and ABM [49]. In this game, users are guided through relaxation techniques (CBT) and must use a glowing light on their headset (their “mind light”), which reacts to activity collected from an electroencephalogram (EEG) they wear during play, to help them navigate the in-game world and defeat monsters they encounter. Nonplayable characters (NPCs) become gradually more difficult to ignore, and players must remain calm (keep their “mind-light” bright) to “decloak” the threats (eg, turn a scary cat into a friendly kitten). The game also rewards players for attending to and quickly responding to positive stimuli and disattending or moving away from negative stimuli (ABM).

A total of 136 children aged between 8 and 13 years (mean 9.93 [SD 1.33]) were selected after screening for elevated anxiety and randomized to either an experimental or control condition. Experimental participants took part in five 1-hour sessions, scheduled twice a week, in which they played the game “MindLight” in groups of 7-19 participants at a time. Control participants undertook the same program in terms of time allocated to game playing, and group size, but the therapeutic game was substituted for a control game “Max and the Magic Marker.” Self- and parent-reported anxiety levels were assessed pre- and postintervention, followed by a 3-month follow-up, using the child and parent versions of the Spence Children’s Anxiety Scale (SCAS-C and SCAS-P) [54]. Latent growth curve modeling revealed a significant slope for all models, indicating levels of anxiety decreased significantly over time. Intention-to-treat linear regression analysis found no significant effect, however, of game condition on anxiety outcome. Qualitative feedback revealed that “MindLight” was more anxiety inducing than “Max and the Magic Marker,” suggesting “MindLight” was successful in achieving its intended emotional exposure effects. Furthermore, no difference was found in perceived difficulty of the 2 games studied, or their perceived appeal to other children. “MindLight,” however, was reported by the participants as less appealing to themselves and less likely to induce flow, a common issue with serious games when they are compared with their more entertainment-focused counterparts [55].

The therapeutic game “Dojo” appeared in 2 of the papers selected for review. “Dojo” is a first-person emotion-management game that takes place in a secret temple below an urban subway. The player assumes the role of a young person experiencing a difficult time, and navigates 3 rooms, headed by a “dojo master” thematically designed to represent different emotions: anger, frustration, and fear. In each room, the “dojo master” trains the player in a relevant coping skill for the emotion, after which the player undertakes a task (eg, in the fear room, the “dojo master” trains the player in deep-breathing exercises), then challenges them to collect bones in a labyrinth while attempting to evade a powerful and frightening angry ghost. The player’s heart rate can be monitored and used by the game to increase or decrease game difficulty.

In one study, “Dojo” was assessed by means of a pilot study using a mixed-methods approach [51]. This study was conducted in 2 residential treatment centers offering 24-hour care for youths with severe mental health problems. A total of 8 adolescents (mean_age_ 14.38 [SD 1.60]) took part in eight 30-min sessions playing “Dojo” on a laptop. These sessions took part twice a week for 4 consecutive weeks—though 3 participants experienced a 2-week break due to scheduling conflicts. The participants rated statements regarding game satisfaction on a 5-point scale, and they were also offered the opportunity to provide comments. The Dutch language version of the SCAS was used to measure anxiety. Reported satisfaction with “Dojo” was high, and both participants and mentors reported high compliance and positive changes in anxiety. The participants did, however, suggest that “Dojo” became repetitive and would have preferred more game rooms.

Following this study, Scholten et al [52] then utilized an RCT design to examine the anxiety-reduction potential of “Dojo” in comparison with “Rayman 2: The Great Escape,” which was chosen as a control game. A total of 138 adolescents (11-15 years, mean_age_ 13.87 [SD 0.91]) were tested both pre- and postintervention, with a 3-month follow-up (N=126) using the SCAS-C. The participants also provided feedback about game experience and game expectations before the intervention. The intervention took place over 3 weeks, consisting of two 1-hour sessions a week. All the participants accessed their games after school hours in the same room regardless of condition, using separate computer terminals and headphones to hear game sound and diminish distractions. Results indicated that anxiety symptoms significantly decreased at follow-up in both conditions (total anxiety symptoms: beta=.70, SE=0.04, *P*<.001; personalized anxiety symptoms: beta=.63, SE=0.05, *P*<.001). Latent growth curve models revealed a steeper decrease of personalized anxiety symptoms in “Dojo,” but not total anxiety symptoms.

Coyle et al [53] studied the therapeutic game “gNats Island” using a series of trials. “gNats Island” is a gamified CBT intervention derived from a paper-based CBT manual for 19 adolescents (total sample aged 11-16 years, 12 males, 7 females). Players navigate a 3D animated tropical island in which they meet a series of NPCs, which introduce mental health concepts using a spoken conversation, embedded animations, videos, and questions regarding the player’s own situation (to which players can respond by a multiple-choice question). Players carry an in-game notebook to answer further questions posed by NPCs and record new ideas. Negative automatic thoughts are represented in-game as “gNats,” which sting players to cause negative thinking. Catching, trapping, and swatting gNats are used to represent identification and challenging of negative thinking.

In one study, therapists independent of the design team used “gNats Island” with adolescents referred to the psychology team at the participating hospital experiencing clinical ADs. Adolescents played the game alongside a clinician who acted as a partner. Results from questionnaire feedback indicated that adolescents found the game more fun and engaging than “just talking,” and assisted in avoiding perceptions of confrontation in direct face-to-face interaction. Quantitative data indicated a decrease in anxiety scores both during and postintervention. No further statistical data are provided in this study.

In a second study, a member of the design team used the game with 15 adolescents experiencing issues including anxiety, but also depression, anger management, and issues relating to autism spectrum conditions. Although some participants used the game in a structured manner, in six 1-hour sessions over 6 weeks, others used the game flexibly as determined by clinician assessments of their individual needs. Qualitative feedback showed that after the intervention stage had finished, the participants preferred to explore the Web-based world, suggesting “winding down” time may be as important as engagement with therapeutic elements of game play. The participants also rated modules 4 and 5 of the game lower than previous elements. Notably, difficulty levels of the game at this point had increased, and a core component of CBT had been introduced, suggesting pacing at this stage of the game may not have been as effective as required. Low graphical fidelity of the game was noted by users, but not reported as a barrier.

The final study eligible for inclusion in this review was conducted by Li et al [50] and investigated the effectiveness of “PlayMotion” hardware (which creates Web-based environments via AR) using a quasi-experimental design. A total of 122 children aged between 8 and 16 years (mean_age_ 11.85 [SD 2.20]) admitted to an oncology ward in a large hospital in Hong Kong were assigned either to experimental (PlayMotion) or control (routine nursing care with no engagement in Web-based interactions) conditions. The participants were recruited in 2 phases, with all patients admitted in phase 1 assigned to control (N=70), followed by a 1-month washout, followed by phase 2, in which all patients admitted were assigned to the experimental condition (N=52). Engagement with the Web-based intervention consisted of 30-min sessions for 5 days a week, with 4 participants per group. Anxiety was measured using the SCAS-C, with results indicating no change in anxiety symptoms after 7 days.

## Discussion

### Principal Findings

Although therapeutic games show early signs of promise in helping to alleviate symptoms of anxiety in adolescent samples, a number of issues and limitations of the extant evidence have emerged. First, although some evidence utilizes RCT protocols to establish a clear comparison between therapeutic games designed specifically for anxiety reduction and control games designed without this primary purpose in mind, other research is based on clinician’s impressions of games, or feedback from adolescents while in the presence of a clinician, who may also be a member of the design team, creating the potential for bias.

Of the RCTs that exist, these are limited in number (N=2) [49,52]. Furthermore, both these studies reported reductions in anxiety in control groups, with no significant differences in anxiety reduction found between the 2 conditions. Authors note that control condition games may have inadvertently utilized game mechanics that trained resilience and coping skills despite this not being their intended or primary purpose [49], or that participants may have vicariously acquired coping strategies from their peers in the experimental group, playing their games in the same room at the same time [49,52].

In addition, as neither study utilized a waiting-list control group or comparison to established nongaming therapy for further comparison, and although the therapeutic games tested seem to have potential, it is difficult in either case, despite their healthy sample sizes, to establish the extent to which specifically designed therapeutic games may have additional capacities for anxiety reduction in adolescents. As other findings offer no follow-up results, offer pre- versus posttest as control [53], or offer a “wait-list” control but use a game designed for multiple conditions [50,53], there is a scope for further research to further examine the capabilities of specifically designed therapeutic games to reduce anxiety in both short term and long term.

Current research has also focused on the evaluation and exploration of the benefits of therapeutic games in relatively controlled environments using hardwares such as EEG, AR hardware, and heart rate monitoring, which are impractical in everyday environments. Intervention sessions in this field are routinely scheduled in classrooms [49,52] or clinical environments, sometimes with a practitioner present to guide the interaction [50,51,53]. Although initial findings suggest that engagement with therapeutic gaming may assist in clinically measurable reductions in anxiety symptoms over time, it is not clear how effective such games may be in real-life environments. It is also unclear how such games may have potential to assist at the point of symptom experience, either as a distraction technique or coping mechanism, in an everyday manner. As highlighted previously, access to, and efficacy of, clinically proven interventions for anxiety is limited by practical and social barriers to treatment [24], lengthy IAPT referrals [26], or inadequate support from their educational institution [27,28]. As a result, as many as 50% of individuals who may benefit do not receive any form of therapy [25], with figures significantly higher for adolescents [29]. Therapeutic games that successfully manage to breach these practical and social barriers to treatment, overcoming the need for hardwares impractical to day-to-day life, may be significant in helping to mitigate the short- and long-term implications of one of the most prevalent psychological disorders in a population that is difficult to treat.

A further theme of the current research concerned the structure and format of delivery of the existing interventions. For instance, in Scholten et al’s [52] RCT to assess “Dojo,” participants reported that the duration of the intervention was “too long,” specifically with regard to maintaining concentration and motivation after approximately 4 sessions. Authors suggested that this was potentially a product of “Dojo” being a small game. Once adolescents finished the available rooms, as most did after 3-4 sessions, they were required to repeat the rooms until the end of the intervention period. Although repeating the rooms may be seen as a reinforcement of their learning, this repetitive play may also have caused boredom. Murphy et al [56] suggest that although some degree of repetition may be beneficial in improving learning and future experience of flow in video games, repetition without learning anything new, or repetition without experience of even subtle changes in gameplay, can disrupt flow and inhibit perceptions of “mastery.” This may explain wider research in the field, which suggests that programs with shorter durations tend to have better outcomes [57].

### Limitations

Despite the initial number of studies found through search terms being extensive, the final number of papers successfully meeting the inclusion criteria was low (N=5). Accordingly, although this was somewhat expected due to the stringency of the criteria used in this review, there is further research using therapeutic games for young adults, which may be of interest in the development of games for anxiety aimed at older adolescents. In addition, while the mean age of participants in papers included in this review was between (or just below) 10-19 years, the studies used individual participants aged below 10. As a result, the data are partially affected by the presence of participants who do not qualify as adolescents, but rather as children, making current research to date problematic in establishing the extent to which currently available therapeutic games may be beneficial for older adolescents on the brink of early adulthood.

Furthermore, this review considered papers with a publication date of up to and including July 2017. Due to the fast-paced nature of technology development, particularly with regard to software applications, of which games are an example, the relevance of this review in terms of its ability to answer the research questions posed may be time-limited.

### Future Research

As noted by authors of papers included in this review, this is an emerging area of interest in the field, and subsequently, there are several avenues of exploration yet to be fully explored. Further research would benefit from full RCT studies ensuring appropriate control games are selected, or by using a waiting-list control as a second control condition, to allow for more rigorous comparison. Furthermore, although some studies to date have utilized a follow-up time point, others have not. As a result, the long-term benefits of the use of therapeutic games for adolescent anxiety is currently unclear.

Future research would also benefit from further consideration of the applicability and efficacy of therapeutic games in more ecologically valid settings. Current research to date has explored the use of therapeutic gaming in systematically controlled environments. Consequently, the potential of using games in day-to-day life or at the point of symptom experience remains unknown. As noted previously, therapeutic games that successfully manage to breach practical and social barriers to treatment, with engaging games capable of repeating concepts of clinical value while maintaining flow, will be of value to the field in establishing the potential of this protocol.

Finally, no current legislative body or code of conduct exists for the development and regulation of “therapeutic games,” nor is there currently a standardized procedure or empirical protocol for their scientific evaluation. In such an unregulated environment, there is substantial potential, therefore, for misuse of the term “therapeutic game” on the part of a more commercially driven developer. The capricious nature of the results presented in the investigations included in this review could be argued to be a product of such methodological variability, rather than an indication of inconsistencies of therapeutic games as effective treatments for anxiety symptoms. Consequently, future research should aim to establish a valid and reliable model for the assessment and verification of therapeutic games, with the view to developing a trustworthy quality-approved protocol.

### Conclusions

This review aimed to assess the effectiveness of therapeutic games in making clinically measurable reductions in AD symptoms in adolescent samples. Although research in this field appears to be extremely limited, as demonstrated by the small number of papers meeting the inclusion criteria for this review, early findings suggest that therapeutic games have potential in helping to reduce anxiety levels in adolescents. By utilizing this protocol in a medium that facilitates overcoming the existing barriers to treatment, therapeutic games may be a valuable facilitator in reducing the short- and long-term implications of one of the most prevalent psychological disorders in a population that is difficult to treat.

## Appendix

Multimedia Appendix 1 Search term definitions and variations of input.

## Abbreviations

ABM: attention bias modification
AD: anxiety disorder
AR: augmented reality
CBT: cognitive behavioral therapy
eHealth: health care practice supported by electronic processes and communication
IAPT: improving access to psychological therapies
mHealth: versions of eHealth using mobile devices
NPC: nonplayable character
RCT: randomized controlled trial
SCAS-C: Spence Children’s Anxiety Scale-Child version
SCAS-P: Spence Children’s Anxiety Scale-Parent version
