# Correction: Empirical Evidence for the Outcomes of Therapeutic Video Games for Adolescents With Anxiety Disorders: Systematic Review

**DOI:** 10.2196/110402

**Published:** 2026-09-04

**Authors:** JMIR Publications Editorial Office

**Affiliations:** 1 JMIR Publications Toronto, ON

Following the publication of “Empirical Evidence for the Outcomes of Therapeutic Video Games for Adolescents With Anxiety Disorders: Systematic Review” [1], concerns were raised regarding a potential conflict of interest between reviewer Amy Rathbone and the authors. These concerns included prior co-publication and shared affiliation with a member of the author list and an active professional relationship between the reviewer and an author. This article has been subsequently reassessed by the editor in chief, who determined that the content of this review did not unduly influence the decision to accept the article. The decision to accept the submission was based primarily on the remaining reviewer’s comments and the editor in chief’s contemporary appraisal of the manuscript.

As such, the JMIR Publications Editorial Office issues this corrigendum to update the Conflicts of Interest statement:

Reviewer Amy Rathbone was found to have an undisclosed conflict of interest with a member of the author list under the terms of JMIR Publications policy. The editor in chief determined that the content of the review did not unduly influence the decision to accept the article. The decision to accept the submission was based primarily on the remaining reviewer’s comments and the editor in chief’s contemporary appraisal of the manuscript.

We regret that this issue was not identified before publication.

The correction will appear in the online version of the paper on the JMIR Publications website together with the publication of this correction notice. Because this was made after submission to PubMed, PubMed Central, and other full-text repositories, the corrected article has also been resubmitted to those repositories.
