# Supplementary material for: Empirical Evidence for the Outcomes of Therapeutic Video Games for Adolescents With Anxiety Disorders: Systematic Review
Source: JMIR Serious Games. 2018 Feb 28;6(1):e3. doi: 10.2196/games.9530 (PMC5852274; doi:10.2196/games.9530)
Supplement: Multimedia Appendix 1 [file games_v6i1e3_app1.pdf]

Appendix 1: Search term definitions and variations of input

The following search terms were used to address the variety of games that might be played, and the variation in terms used to describe them: all(‘serious game’ OR ‘video game’ OR ‘therapeutic game’ OR ‘online game) AND all(‘adolescen*’ OR ‘teenage’ OR ‘youth’ OR ‘young adult*’) AND anxi*’

Game-related keywords	Sample-related keywords	Anxiety-related keywords
**Serious Game**<br>**Defined as ‘a game designed with a primary purpose other than for entertainment.’**	Adolescen*<br>Defined using APA criteria (10-19years).	Anxi*<br>Group of mental disorders characterized by significant feelings of anxiety and fear. Anxiety is a worry about future events and fear is a reaction to current events, with physiological markers such as high heart rate.
**Video Game**<br>**Defined as a game played via electronic mediums such as computers/consoles or mobile devices.**	Teenage<br>Aged between 13-19. Alternative word for ‘adolescent’.	
**Therapeutic Game**<br>**Defined as a game designed for the delivery of psychological therapy.**	Youth<br>Term used to describe young people and as alternative for adolescent.	
**Online Game**<br>**Defined as a game played via the Internet.**	Young Adult<br>Term used to describe older adolescents or individuals in early years of adulthood.	

Variation of input

Peer-reviewed publications were search using the general search structure, and included in the review subsequent to meeting the inclusion criteria.
